# Carbon footprint reduction associated with a surgical outreach clinic

**DOI:** 10.1186/s40463-021-00510-4

**Published:** 2021-04-19

**Authors:** David Forner, Chad Purcell, Victoria Taylor, Christopher W. Noel, Larry Pan, Matthew H. Rigby, Martin Corsten, Jonathan R. Trites, Antoine Eskander, Ted McDonald, S. Mark Taylor

**Affiliations:** 1 Division of Otolaryngology - Head & Neck Surgery, Dalhousie University, Halifax, Nova Scotia Canada; 2 Department of Otolaryngology – Head & Neck Surgery, University of Toronto, Toronto, Ontario Canada; 3 Department of Radiation Oncology, Dalhousie University, Halifax, Nova Scotia Canada; 4 Department of Radiation Oncology, Queen Elizabeth Hospital, Charlottetown, Prince Edward Island Canada; 5 Department of Otolaryngology – Head & Neck Surgery, Sunnybrook Health Sciences Centre, Toronto, Ontario Canada; 6 Department of Economics, University of New Brunswick, Fredericton, New Brunswick Canada

**Keywords:** Head and neck neoplasms, Carbon footprint, Outreach

## Abstract

**Background:**

Healthcare systems generate substantial carbon footprints that may be targeted to decrease greenhouse gas emissions. Outreach clinics may represent tools to assist in this reduction by optimizing patient related travel. Therefore, we sought to estimate the carbon footprint savings associated with a head and neck surgery outreach clinic.

**Methods:**

This study was a cross-sectional survey of patient travel patterns to a surgical outreach clinic compared to a regional cancer treatment centre from December 2019 to February 2020. Participants completed a self-administered survey of 12 items eliciting travel distance, vehicle details, and ability to combine medical appointments. Canadian datasets of manufacturer provided vehicular efficiency were used to estimate carbon emissions for each participant. Geographic information systems were used for analyses.

**Results:**

One hundred thirteen patients were included for analysis. The majority of patients (85.8%) used their own personal vehicle to travel to the outreach clinic. The median distance to the clinic and regional centre were 29.0 km (IQR 6.0–51.9) and 327.0 km (IQR 309.0–337.0) respectively. The mean carbon emission reduction per person was therefore 117,495.4 g (SD: 29,040.0) to 143,570.9 g (SD: 40,236.0). This represents up to 2.5% of an average individual’s yearly carbon footprint. Fewer than 10% of patients indicated they were able to carpool or group their appointments.

**Conclusion:**

Surgical outreach clinics decrease carbon footprints associated with patient travel compared to continued care at a regional centre. Further research is needed to determine possible interventions to further reduce carbon emissions associated with the surgical care of patients.

**Graphical abstract:**

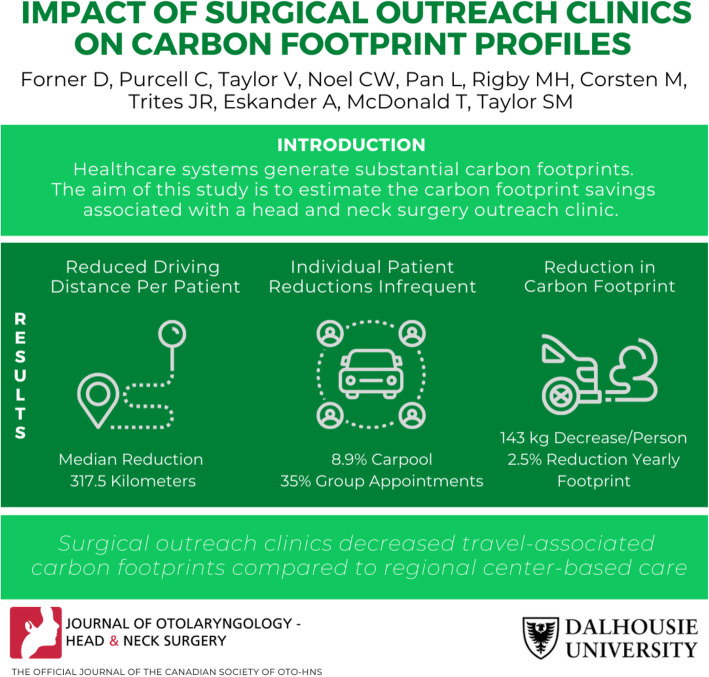

**Supplementary Information:**

The online version contains supplementary material available at 10.1186/s40463-021-00510-4.

## Introduction

Global warming and climate change represent a significant danger to the environment and human health. Driven in large part by human urbanization and emission of greenhouse gases, global warming is predicted to raise the Earth’s ambient temperature by up to 5 °C by 2050 and have a significant impact on economies and health [1].

In order to study individual and large group contributions to emissions and climate change, the idea of the carbon footprint was put forth [1]. Healthcare provision and large institutions contribute substantially to the overall carbon footprint, often accounting for a tenth or more of a country’s total emissions [2]. Almost half of healthcare related emissions are from the hospitals themselves, including up to 15% for electricity alone [3], while a large amount also comes from the pharmaceutical industy [4]. There are many avenues through which alterations in healthcare provision and operating procedures can be capitalized on to reduce the carbon footprint. Optimized energy sources, reduction in water related waste, and reduced packaging are a few amongst many [5–7].

Outreach clinics and mobile health units have been shown to improve health outcomes and be cost effective [8]. These interventions may take multiple forms, ranging from providing outpatient services in completely non-serviced areas, to specialist inclusion on liaison teams from large regional centres to those that have no sub-specialists locally. Previously described benefits of outreach clinics have included reduced patient travel time and costs, improved provision to guideline adherent medical care, and improved patient outcomes [8]. Interestingly, while some services have shown an intent to reduce the carbon footprint associated with healthcare travel [9, 10], there are no studies that assess the impact of outreach clinics on the reduction of carbon footprints.

Therefore, we sought to estimate the carbon footprint reduction associated with a head and neck surgical oncology outreach clinic, as well as determine current system- and patient-level practices for mitigating travel related emissions.

## Methods

### Study design

This study was a cross-sectional survey. Patients were recruited during their head and neck oncology clinic appointments at the Prince Edward Island (PEI) Cancer Treatment Centre (PEICTC) in Charlottetown, PEI over three separate occasions from December 2019 to February 2020. The Queen Elizabeth II Health Sciences Centre (QEII HSC) in Halifax, Nova Scotia is a tertiary academic centre that provides regionalized advanced head and neck surgical oncology services for the majority of the Canadian Maritime provinces. The PEICTC is located approximately 325 km from the QEII HSC. Operative procedures for all consulted patients are carried out at the QEII HSC, regardless of province of residence. The senior investigator (SMT), an attending surgeon at the QEII HSC, holds a surgical outreach clinic at the PEICTC for new consultations and ongoing oncology surveillance. Therefore, patients living in the province of PEI are able to access aspects of care intra-provincially. Pathology treated includes upper aerodigestive tract, thyroid, salivary gland, and cutaneous malignancies of the head and neck. Only the surgeon travels from the QEII HSC to the PEICTC as all other necessary staff, for instance nursing and administrative supports, are local PEICTC staff.

Eligible PEI residents were enrolled and asked to complete a survey regarding their travel to the surgical outreach clinic along with previous or expected travel to the regional Halifax cancer treatment centre (QEII HSC). Each participant completed the survey once.

### Ethics & Eligibility

This study was reviewed and approved by the Prince Edward Island Research Ethics Board.

Inclusion criteria included: adult patients (age > 18 years old) and patients presenting as a new consultation or for follow-up surveillance under the care of the senior author (SMT). Patients were excluded if they did not travel by motor vehicle or if they were unable to provide their own informed consent.

### Outcomes

The primary outcome of interest was the difference in carbon emission associated with observed travel to the surgical outreach clinic and the expected travel to the regional cancer centre. The secondary outcomes of interest included assessment of sustainability practices including grouping of medical appointments and carpooling to clinic visits.

### Survey

Each participant was given a two-part survey with a total of 12 items. Part I pertained to travel from the residence of the patient to the surgical outreach clinic (Supplemental Fig. 1). The questions involved the mode of transportation, the make, model and year of the vehicle, and questions relating to carpooling and grouping of medical appointments. Part II concerned travel from the residence of the patient to the Halifax regional head and neck oncology clinic. The questions were answered based on whether or not the participants had previously travelled to Halifax for head and neck oncology appointments. If yes, how they did travel; if no, how would they expect to travel. Surveys were self-administered and assisted by a member of the research team when necessary. As few patients ultimately chose the option of ferry travel, all patients were considered to have driven to Halifax based appointments and carbon emissions were calculated in an identical manner to those who chose the driving option alone.

Pre-testing of the survey was performed to ensure that survey questions were clear and correctly interpreted by study participants [11]. The first five participants were interviewed by a research assistant (VT) in a semi-structured format while they completed the survey (Supplemental Table 1). The research assistant prompted the patients to verbalize their thoughts and opinions of each item in order to determine if participants interpreted the items as the investigators intended. Pre-testing revealed that participants did not have any trouble understanding the survey, therefore no changes were made.

### Carbon footprint estimation

Fuel consumption rate datasets for motorized vehicles from 1995 to 2020 were accessed through Natural Resources Canada [12]. These datasets contain the make, model and year of vehicles with their associated fuel consumption rates (L/100 km). These rates were generated using a combination of data from vehicle manufacturers that incorporate standard laboratory testing and procedures to estimate the fuel consumption rates of their models [13]. The product of the combined fuel consumption rate (L·100 km^− 1^) of each vehicle and the amount of CO_2_ generated per litre of fuel (2300 g·L^− 1^) yields the carbon dioxide (CO_2_) generated per kilometer (g·km^− 1^) of travel; these values are provided in each dataset and were used to calculate the carbon footprint as a function of distance travelled (below). Two additional variables are used to calculate specific carbon emission yields but were unavailable in this study (engine size and number of cylinders). To accommodate this uncertainty, both the lowest and highest available carbon emissions for each make, model, and year were calculated and ranges are provided.

The postal code of each participant and the postal code of the surgical outreach clinic (PEICTC) were entered into Google Maps to calculate distance travelled (observed distance). This was repeated using patient postal codes and the postal code of the regional cancer centre (QEII HSC, expected distance). Return trip distances travelled by each patient (ie, to and from destinations) were used to calculate and compare the total CO_2_ emissions (grams) generated by their vehicles as a product of distance (kilometers) and carbon emission efficiency (g·km^− 1^). The average total annual carbon emission savings was derived by multiplying the average carbon emission difference per person by an expected number of patients per three-month period (approximated as 100 patients) and multiplying by four.

### Analysis

Continuous variables are presented as mean (standard deviation (SD)) or median (interquartile range (IQR)). Normality was assessed through the Shapiro-Wilk test and visual inspection of histograms and Q-Q plots. Categorical variables are presented as counts and relative frequencies. Distances were further visualized through the creation of a straight-line map using a geographic information system. All analyses were performed using Microsoft® Excel for Mac (version 16.35, Microsoft, Redmond, Washington, USA), SAS University Edition 2.8 9.4 M6 (SAS Institute, Cary, North Carolina, USA), and a Geographic Information System (ArcGIS Online, Esri, Redlands, California, USA).

## Results

### Demographics & survey

All potentially eligible patients that were approached for enrollment in the study agreed to participate, resulting in a recruitment rate of 100%. In total, 118 patients were recruited, and 113 patients returned survey data in a usable form (completion rate 95.8%). All excluded patients (*N* = 5) were due to an inability to clarify survey item answers related to the primary outcome. These instances were due to either multiple answers given to single-answer items or answers being written as free-text outside of the provided response boxes. Pre-testing of the survey identified no issues requiring revision of the survey. No patients (0%) indicated that they had trouble understanding the survey. The mean age was 64.8 years (SD: 13.2), the majority of participants were male, and many patients were of low socioeconomic status as defined as geocoded dissemination areas [14] (Table 1).

**Table 1:** Demographics of participating patients

Variable	Values
**Age (mean years, SD)**	64.8 (13.2)
**Gender (N, %)**	
Male	76 (67.3)
Female	37 (32.7)
**Income Quintile (N, %)**^a^	
1 (Lowest)	25 (23.4)
2	38 (35.1)
3	25 (25.2)
4	13 (12.5)
5 (Highest)	3 (2.8)
Missing^b^	2 (1.9)

^a^Denominator = 107 (number of patients used for subsequent carbon footprint analysis)

^b^Missing values due to inability to appropriately perform geocoding

### Primary outcome – carbon emission

The majority of patients used their own vehicle for transportation to the outreach clinic (Table 2). One hundred seven patients (94.7%) used their own personal vehicle or carpooled with other people and were therefore included for estimation of the carbon footprint associated with travelling to the surgery outreach clinic. Of the six participants that did not carpool or travel in their own vehicle, four used a taxi and two took a shuttle or bus.

**Table 2:** Mode of Transportation used by participants when travelling to the surgical outreach clinic

Item	N (%)
**Mode of Transportation**	
Carpool or Other’s Vehicle	10 (8.85)
Own Vehicle	97 (85.84)
Shuttle or Bus	2 (1.77)
Taxi	4 (3.54)
**Accompaniment**	
No	54 (47.8)
Yes	59 (52.2)
**Relationship if Accompanied**	
Family	19 (32.8)
Friend	1 (1.7)
Partner/Spouse	38 (65.5)
**Grouped Appointments (Self)**	
No	69 (62.2)
Yes	39 (35.1)
Unclear	3 (2.7)
Missing	2
**Other Specialists**	
No	107 (94.7)
Yes	6 (5.3)
**How Travel to Halifax**	
Unclear	1 (1.11)
Carpool or Other’s Vehicle	14 (15.56)
Own Vehicle	70 (77.78)
Shuttle or Bus	5 (5.56)

**Fig. 1:**
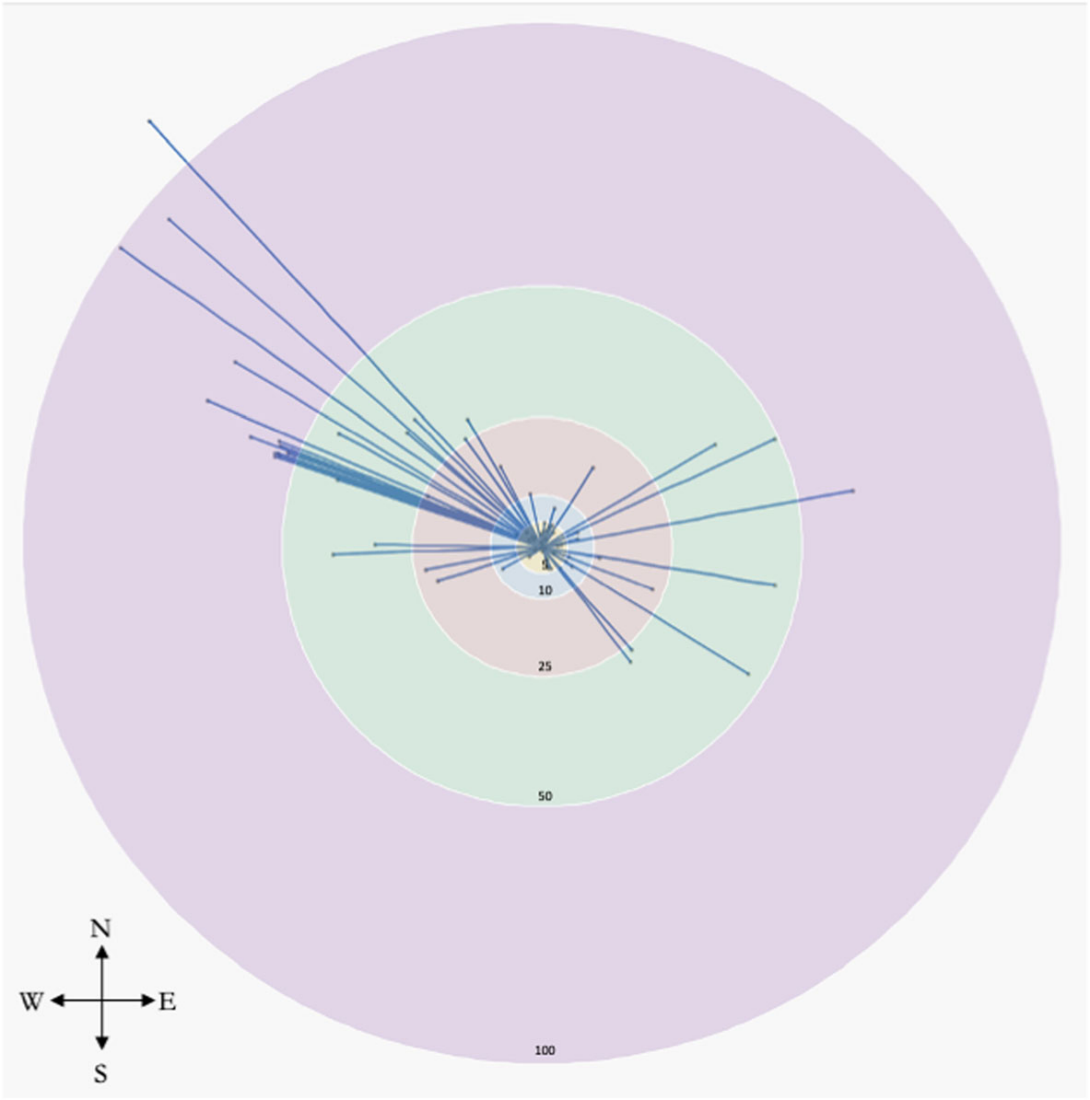
Patient travel to the surgical outreach clinic visualized as straight-line travel created through a Geographic Information System (GIS) mapping platform. The central point where all straight lines converge represents the clinic location. The inner most circle has a 5 km radius, followed by 10 km, 25 km, 50 km, 100 km and > 100 km. Actual driving distances are expected to be of greater distances

The median distance from participants homes to the surgery outreach clinic was 29.0 km (IQR 6.0–51.9; Fig. 1), while the median distance to the regional centre was 327.0 km (IQR 309.0–337.0; Fig. 1). This yielded a median difference of 317.5 km (IQR 250.2–325.6). One fifth (*N* = 23, 20.4%) of patients lived within 5 km of the outreach clinic, and three quarters were within 50 km (*N* = 84, 74.3%). Only five patients travelled more than 100 km (4.4%).

**Fig. 2:**
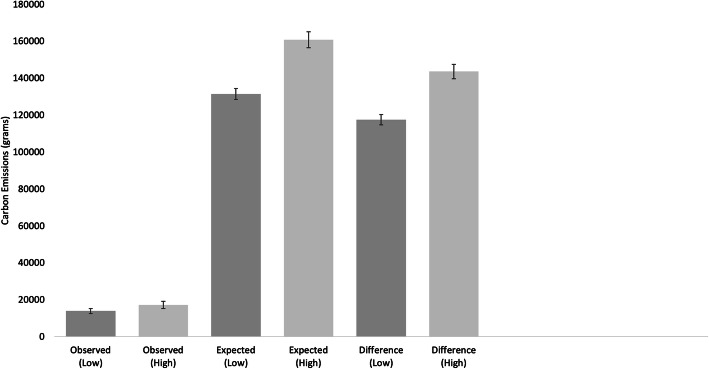
The observed (associated with travel to the outreach clinic) and expected (associated with potential travel to the regional centre) per patient carbon emissions, including both high (right, light grey) and low (left, dark grey) estimates (see text) and differences between observed and expected. Error bars represent the standard error

Carbon emission efficiency of all vehicles varied between the low and high estimates provided, with means of 199.6 g·km^− 1^ (SD: 43.4) and 243.6 g·km^− 1^ (SD: 61.6) respectively. The observed (travelling to the surgical outreach clinic) and expected (travelling to the regional centre) carbon emissions were estimated as a product of driving distance and efficiency values. The median observed low estimate of carbon emission was 10,411.2 g (IQR: 2267.2–21,254.4) and the expected estimate was 130,082.0 g (IQR: 107,724.0 – 149,960.0). This yielded a mean carbon emission difference of 117,495.4 g (SD: 29,040.0; Fig. 2). Likewise, the high estimate yielded a saved carbon emission of 143,570.9 g (SD: 40,236.0; Fig. 2). Extrapolating this three-month period to an annual basis would yield an approximate total mean carbon emission savings of 46,998,160 g.

The total distance travelled by the attending surgeon, taken as the round-trip distance between the regional cancer centre and the surgical outreach clinic, was 330.0 km. The carbon emission efficiency was 211 g·km^− 1^, yielding a carbon footprint of 69,630 g per clinic held. Across the time period of this study, three clinics were held, amounting to 208,890 g of carbon emitted.

### Secondary outcome – sustainability practices

Over half of the participants were accompanied by another individual during travel to the appointment, of which the majority were self-indicated to be a partner or spouse (Table 2). Thirty-five percent of patients indicated they grouped their own medical appointments together when possible, and 5.3% (*N* = 6) saw other specialists the same day. However, only 8.9% (*N* = 10) indicated they were carpooling for the purposes of grouping medical appointments with their travel companion (Table 2).

Of those participants who indicated they had travelled to the regional centre for treatment already (*N* = 89), the majority did so via their own vehicle (Table 2). Only a minority indicated the use of a shuttle, bus service, or carpooling. In those participants who had not yet needed to travel to the regional centre for treatment of their head and neck cancer, the majority (78.3%) indicated they would travel in their own car; only one participant indicated they would use a shuttle or bus service.

## Discussion

There is a substantial carbon footprint related to traveling to healthcare appointments. This study has shown carbon emissions may be reduced by providing outreach clinics when healthcare is regionalized. While this finding is intuitive, this is the first study to our knowledge that estimates the magnitude of carbon emission reduction associated with outreach clinics. As well, this study has shown carpooling and combining medical appointments occurs infrequently compared to singular travel and appointment making.

The logistics of the outreach clinic examined in this study allow for substantial carbon footprint savings. Only a single surgeon travels to the clinic from the regional centre; all required support staff are local and thus have minimal travel needs. As such, saving one patient from travelling to the regional centre results in a carbon neutral scenario when considering travel alone. Over 50 patients are seen at each outreach clinic, thus facilitating large carbon reductions. There are unmeasured carbon costs that must be considered, such as the procurement of supplies necessary to hold the clinic. However, the majority of endoscopes and related instruments were already possessed by the outreach clinic facility, and thus these procurements are thought to be minimal.

The per person reduction found in this study amounts to 0.5 to 2.5% of an average individual’s yearly carbon footprint [15]. It must be recognized that a small proportion of this benefit is mitigated by the travel of the attending surgeon to the surgical outreach clinic. Similar to other avenues of healthcare provision, the outreach clinic itself has associated carbon footprint costs that were not examined here. However, these could be assumed to be relatively approximate of those generated if patients were seen at the regional centre.

Regionalization of cancer treatment to a few high volume centres has proven benefits in patient-related outcomes, including overall survival [16]. As an increasing proportion of the population becomes urbanized, regionalization of care will only continue to grow [17]. However, the implications of this regionalization on patient and caregiver travel burden should be considered, including the unintended consequences such as increased carbon footprint.

Many countries have committed to reducing greenhouse gas emissions through participation in the Paris Agreement. Canada has set a target to reduce its carbon footprint 30% below 2005 levels by 2030 [18]. The Climate Change Act in the United Kingdom seeks to reduce carbon emissions by 80% below 1990 levels. Healthcare associated emissions are a major contributor to overall emissions [19] and up to 20% of healthcare associated emissions may in turn be attributable to personal travel of employees and patients [20]. Few studies have quantified this burden any further [21, 22].

The applications of telehealth are widespread, including in the realm of reducing carbon emissions through resource intensive travel methods [23]. However, in some situations, telehealth approaches are still lacking and cannot accommodate all requirements of complex patient interactions. The diagnosis and subsequent post-treatment surveillance of head and neck cancer is one such instance and this has been particularly highlighted during the COVID-19 pandemic period during which this manuscript was submitted. Telehealth options have been reported [24, 25], but few show outcomes related to lack of endoscope surveillance or monitoring by less skilled endoscopists. Full assessment of the oral cavity and oropharynx requires careful illumination and palpation neither of which are possible via telehealth. Similarly, the neck examination is limited to inspection which is less than adequate. Therefore, in-person follow-up remains a key component to high quality head and neck cancer care.

Additional benefit beyond that of improved patient care and reduced carbon footprints is that of a reduction in out-of-pocket expenses by patients. Surgical outreach clinics in Canada and elsewhere have shown a reduction in patient associated costs [26, 27]. This is especially important in a head and neck cancer population that is often marginalized and of low socioeconomic status, but nonetheless faces significant financial toxicity associated with treatment [28, 29]. This is possibly observed in our patient population given the relatively low socioeconomic status found. Opportunity costs are also improved with outreach clinics as time off work, need for overnight accommodations, and other related expenses are reduced. Although beyond the scope of this study, it should also be noted that patient preference should be incorporated into care models. Patients prefer to be seen closer to home, particularly for rural patients, as this leads to less travel burden and increased access to transportation support from family and friends.

The findings of this study must be interpreted within the context of its design. Although relatively few participants indicated they were able to group their medical appointments together or with other individuals, social desirability may be present, as patients were aware the study investigated the carbon footprint associated with healthcare. Estimates of the magnitude of carbon emissions should be considered approximate, as measurement error could have resulted from improper recall of vehicle model, make, or year. As well, carbon emissions are dependent on engine size and number of cylinders, which we were unable to adequately elicit from patients. In order to account for this uncertainty, ranges of carbon emissions were calculated and, within the variances incorporated, would not change our main findings or conclusions. For simplification, the combined fuel consumption rating, rather than the highway specific rating, was used for calculations. Real world driving is likely to result in more emissions than those provided under “ideal” testing conditions, and therefore this trade-off is likely reasonable. Additionally, while the survey was indicated to be well understood by all patients and there was no adjustment needed from the pre-test phase, patients nonetheless provided unclear answers in a minority of cases. Lastly, it can be difficult to visualize the impact by grams of carbon emissions saved. In addition to comparing our findings to the reduction in individual yearly carbon footprint, it may also be helpful to think of these savings as up to 50 servings of beef, over 100 servings of fish, and large amounts of grain crops saved per person [30]. When extrapolated across an annual series of outreach clinics, this amounts to savings equivalent to almost 10 Canadian household’s worth of yearly electricity consumption [31]. Clearly, the implications across an entire clinic roster quickly amount to appreciable reductions.

Strengths of this study include its novel positioning within the healthcare and sustainability literature in the examination of surgical outreach clinics and their associated carbon footprints. The utilization of Google Maps algorithms and actual driving distance, as opposed to “as-the-crow-flies,” also allowed for a more accurate estimation of distances to healthcare contact. However, routes may change depending on additional stops, detours, etc., and so our calculations may represent underestimations of the true distance. This study also represents the majority of patients in the roster of the outreach clinic, with high completion rate, and participants were recruited on multiple dates throughout the study period, potentially reducing selection bias and increasing generalizability of our findings.

Intrinsic to the nature of cancer treatment and surveillance is the severity of disease. Patients with more advanced cancer often require more frequent follow-up for longer time horizons, and also require treatment by a multidisciplinary team [32, 33]. This study did not account for frequency of follow-up which would impact carbon emissions substantially. Future prospective studies should investigate the impact of visit frequency and interventions, as well as the impact of healthcare professionals and their travel, to reduce carbon footprints even further. As well, additional studies in other healthcare systems would aid generalizability of this single surgeon study in a moderate sized region. While health services benchmarks such as wait times are similar for the outreach clinic compared to the regional centre, additional work on clinical outcomes is also required.

## Conclusion

There was a drastic reduction in the carbon footprint of patients travelling to an intra-provincial outreach clinic compared to an inter-provincial regional centre. Further work must be done to reduce the emissions produced by traveling to the outreach clinic itself, as well as to necessary regional centres. Promotion of public transport, use of pre-arranged combined medical appointments, and other interventions would help achieve a goal of further reducing carbon emissions associated with healthcare.

## Supplementary Information

**Additional file 1: Supplemental Figure 1.** Survey used. **Supplemental Table 1.** Semi-structured interview questions for survey pre-test.

## Data Availability

Data is available upon specific request to the corresponding author.
