# Supplementary material for: Carbon footprint reduction associated with a surgical outreach clinic
Source: J Otolaryngol Head Neck Surg. 2021 Apr 19;50:26. doi: 10.1186/s40463-021-00510-4 (PMC8054848; doi:10.1186/s40463-021-00510-4)
Supplement: Supplementary file 1 — Additional file 1: Supplemental Figure 1. Survey used. Supplemental Table 1. Semi-structured interview questions for survey pre-test. [file 40463_2021_510_MOESM1_ESM.docx]

**SUPPLEMENTAL MATERIAL**

**Supplemental Figure 1.** Survey used

|  | | **PART I: TRAVEL FROM HOME TO**  **THE HEAD & NECK ONCOLOGY CLINIC AT PEICTC** | | |
| --- | --- | --- | --- | --- |
| **QUESTION 1** | **When travelling to the PEI Cancer Treatment Centre in Charlottetown, what was your mode of transportation?** | | |  |
|  | ☐ Own Vehicle (Car, Truck, Van, SUV etc.)  ☐ Shuttle or Bus  ☐ Carpool/Other’s vehicle  ☐ Taxi | | |  |
| **QUESTION 2** | **If you traveled in a vehicle, please fill out the following information with regard to your vehicle** | | |  |
|  | **Make**  **(ie: Ford)** |  | |  |
|  | **Model**  **(ie: F150)** |  | |  |
|  | **Year** |  | |  |
| **QUESTION 3** | **Did you travel with anyone else to the PEI Cancer Treatment Centre?** | | |  |
|  | ☐ Yes  ☐ No | | |  |
| **QUESTION 4** | **If you answered YES to QUESTION 4, what is their relation to you?** | | |  |
|  | ☐ Family Member  ☐ Partner/Spouse  ☐ Friend  ☐ Other | | |  |
| **QUESTION 5** | **Do you group medical appointments in Charlottetown so they all can be done with one trip?** | | |  |
|  | ☐ Yes  ☐ No | | |  |
| **QUESTION 6** | **In general, do you group your medical appointments in Charlottetown with other people from around your home?** | | |  |
|  | ☐ Yes  ☐ No | | |  |
| **QUESTION 7** | **Did the people you traveled with also have medical appointments in Charlottetown?** | | |  |
|  | ☐ Yes  ☐ No | | |  |
| **QUESTION 8** | **Are you seeing any other medical specialists at the Queen Elizabeth Hospital during this trip?** | | |  |
|  | ☐ Yes  ☐ No | | |  |
|  | | **PART II: TRAVEL FROM HOME TO**  **HALIFAX FOR HEAD AND NECK ONCOLOGY APPOINTMENTS** | | |
| **QUESTION 9** | **Have you ever travelled to Halifax for a head and neck oncology appointment?** | | |  |
|  | ☐ Yes ☐ No | | |  |
| **QUESTION 10** | **Complete this column if you HAVE travelled to Halifax for a head and neck oncology appointment** | | **Complete this column if you HAVE NOT travelled to Halifax for a head and neck oncology appointment** |  |
|  | **How DID you travel if you had a head and neck oncology appointment in Halifax?** | | **How WOULD you travel if you had a head and neck oncology appointment in Halifax?** |  |
|  | ☐ Own Vehicle (Car, Truck, Van, SUV etc.)  ☐ Shuttle or Bus  ☐ Carpool/Other’s vehicle  ☐ Taxi | | ☐ Own Vehicle (Car, Truck, Van, SUV etc.)  ☐ Shuttle or Bus  ☐ Carpool/Other’s vehicle  ☐ Taxi |  |
| **QUESTION 11** | **If you answered YES to Question 9, How many times have you used the Confederation Bridge or PEI Ferry when travelling to your Halifax for a head and neck oncology appointment ? (please circle)** | | **If you answered NO to Question 9**  **How WOULD you have travelled to Halifax? (please circle)** |  |
|  | **BRIDGE**  **0 1 2 3 4 5 6 7 8 9 10 +**  **FERRY**  **0 1 2 3 4 5 6 7 8 9 10 +** | | **BRIDGE**  **FERRY** |  |
| **QUESTION 12** | **Did you have any trouble understanding the questionnaire?** | | |  |
|  | ☐ Yes  ☐ No | | |  |

**Supplemental Table 1.** Semi-structured interview questions for survey pre-test.

| Semi-Structured Interview Questions |
| --- |
| 1. Did you have any trouble understanding the questionnaire? |
| 1. What did you think question 1 was asking? Question 2….3….12? |
| 1. Is there anything you would change to make the questionnaire more clear? |
